# Meta-analysis of non-tumour doses for radiation-induced cancer on the basis of dose-rate

**DOI:** 10.3109/09553002.2010.545862

**Published:** 2011-01-21

**Authors:** Hiroshi Tanooka

**Affiliations:** Radiation Effects Association, 1-9-16 Kaji-cho, Chiyoda-ku, Tokyo, and National Institute of Radiological Sciences, 4-9-1 Anagawa, Inage-ku, Chiba, Japan

**Keywords:** radiation cancer risk, non-tumour dose, dose-rate

## Abstract

*Purpose:* Quantitative analysis of cancer risk of ionising radiation as a function of dose-rate.

*Materials and methods:* Non-tumour dose, D_nt_, defined as the highest dose of radiation at which no statistically significant tumour increase was observed above the control level, was analysed as a function of dose-rate of radiation.

*Results:* An inverse correlation was found between D_nt_ and dose-rate of the radiation. D_nt_ increased 20-fold with decreasing dose-rate from 1-10^−8^ Gy/min for whole body irradiation with low linear energy transfer (LET) radiation. Partial body radiation also showed a dose-rate dependence with a 5- to 10-fold larger D_nt_ as dose rate decreased. The dose-rate effect was also found for high LET radiation but at 10-fold lower D_nt_ levels.

*Conclusions:* The cancer risk of ionising radiation varies 1000-fold depending on the dose-rate of radiation and exposure conditions. This analysis explains the discrepancy of cancer risk between A-bomb survivors and radium dial painters.

## Introduction

The dose-rate of ionising radiation that humans have been exposed to from natural to accidental radiation sources varies over a wide range from 10^−9^ to 10^7^ Gy/min. Radiation dose-rate affects the magnitude of cancer risk even for the same total dose, and in addition changes the shape of the dose-response curve. For assessment of cancer risks of ionising radiation resulting from different exposure conditions, ideally, a set of dose response curves is needed for each dose-rate.

Currently, the estimation of human cancer risk from low doses of radiation is an important problem and data have been extensively reviewed (Committee on the Biological Effects of Ionizing Radiation [BEIR]/National Research Council [NRC], United Nations Scientific Committee on the Effects of Atomic Radiation [UNSCEAR] 1986, 2000, National Council on Radiological Protection and Measurements [NCRP] 1980, BEIR V 1990, BEIR VII 2005, National Radiological Protection Board [NRPB] 1995, Duport 2003). The dose and dose-rate effectiveness factor (DDREF) for cancer risk was determined as 2-10 depending on the target organ (NCRP 1980, International Commission on Radiological Protection [ICRP] 1991, UNSCEAR 1993, NRPB 1995). The application of the linear non-threshold (LNT) model, based on the apparently linear dose-response relation of cancer mortality obtained from extremely high dose-rate cases of A-bomb survivors, was recommended for the estimation of the cancer risk of low dose radiation for protection purposes (NCRP 2001, Brenner et al. 2003, BEIR VII 2005, ICRP 2006); however, the LNT model was questioned for its validity from experimental and epidemiological evidence (Kondo 1993, Academie des Sciences 1997, Tanooka 2001, Tubiana et al. 2006, Feinendegen et al. 2007). The history of the LNT model explains how the idea of a tolerance dose was changed to the linearity concept by incorporating the view of the geneticist (Calabrese 2009). However, a recent review of new biological and epidemiological data still adopted the LNT model (Mullenders et al. 2009). Whatever the model, there exists both linear and threshold type dose-response relations for radiation-induced cancers in experimental and epi-demiological data. For example, the shape of the dose-response curve for cancer incidence may conform to a linear type for leukemia and solid cancers in A-bomb survivors (Chomentowski et al. 2000), while it is non-linear, or even threshold-like, for bone tumours in radium dial painters (Rowland et al. 1978) and liver tumours in thorotrast-injected patients (Anderson and Storm 1992). This discrepancy remained still to be explained.

In a previous study, non-tumour dose, D_nt_, was defined as the highest dose at which no statistically significant tumour increase was observed above the control level. It was proposed as a measure of the upper limit of radiation dose for non-detectable cancer and D_nt_ values were surveyed for in the literature. The results showed that D_nt_ depended on exposure conditions, i.e., acute, protracted, and chronic exposures for whole body and partial body radiation for either low linear energy transfer (LET) or high LET radiation, respectively, with an inverse correlation between D_nt_ and dose-rate (Tanooka 2001). The present study aimed to show the dose-rate dependence of D_nt_ more quantitatively as a function of the dose-rate of radiation.

### Data base

Dose-response data covering ionising radiation exposures from non-tumour to tumour-inducing doses were surveyed in the literature and are listed in Table I. These include D_nt_ values, and corresponding dose-rates of radiation in mice, rats, dogs, and humans with different tumour types obtained under different exposure conditions. Data in the previous study (Tanooka 2001) and additional data were used for the present quantitative analysis. The data numbers in the previous study were unchanged for the convenience of comparison.

**Table I:** Dose-rate of radiation and non-tumour dose, D_nt_.

Data number	Subject		Radiation^a^	Tumour	Dose-rate, Gy/min	Non-tumour dose D_nt_, Gy	Reference
I. Acute exposure							
1	Mouse	RFM/Un	WB γ-ray	thymic lymphoma	0.45	0.1	Ullrich et al. (1976)
2	"	"	"	Harderian tumour	0.45	0.1	"
3	"	"	"	uterine tumour	0.45	0.25	"
4	"	"	"	mammary tumour	0.45	0.25	"
5	"	"	"	myeloid luekemia	0.45	0.25	Ullrich & Storer (1979a)
6	"	"	"	reticulum cell sarcoma	0.45	*>3	Ullrich et al. (1976), Ullrich & Storer (1979a)
7	"	"	"	ovarian tumour	0.45	0.1	Ullrich et al. (1976), Ullrich & Storer (1979b)
8	"	"	"	pituitary tumour	0.45	0.25	"
9	"	"	"	lung adenoma	0.45	2	"
10	"	"	"	thymic lymphoma	0.45	0.1	Ullrich & Storer (1979c)
11	"	"	WB γ-ray, protracted	"	5.8×10^−5^	0.5	"
12	"	"	"	ovarian tumour	5.8×10^−5^	0.5	"
13	"	"	PB X-ray	lung adenoma	4	2.5	Ullrich et al. (1979)
14	"	"	PB neutron	"	5×10^−2^	0.1	"
15	"	BALB/c	WB γ-ray	lung adenocarcinoma	0.4	0.1	Ullrich (1983)
16	"	"	"	ovarian tumour	0.4	0.1	"
17	"	"	WB fission neutron	"	5×10^−2^	0.025	"
18	"	"	WB ^252^Cf neutron	"	7×10^−5^	0.05	Ullrich (1984)
19	"	"	WB γ-ray	thymic lymphoma	4	2	Maisin et al. (1983)
20	"	BC3F1	WB X-ray	hepatocellular carcinoma	1.3	0.5	Di Majo et al. (1986)
21	"	"	"	solid tumour, malignant lymphoma	6×10^−2^	0.64	Covelli et al. (1988)
22	"	"	WB neutron	"	1.7×10^−5^	0.04	"
23	"	Swiss	PB electron	skin tumour	5.5	0.8	Albert et al. (1972)
24	"	CBA/H	PB β ray fractionated	"	5.5, split	*60	Hulse & Mole (1969)
25	Rat	WAG/Rij	WB γ-ray, fractionated	mammary carcinoma	4×10^−4^	1	Bartsra et al. (2000)
26	"	Long-Evans	PB X-ray	thyroid adenoma	2.5	1	Lee et al. (1982)
27	"	Sprague-Dawley CD	PB β-ray	skin tumour	5	10	Burns et al. (1975, 1993)
28	"	"	PB electron	"	5, split	*20	Burns et al. (1975)
29	"	"	PB proton	"	1.38	0.75	Burns et al. (1978)
30	Human	A-bomb survivor	WB γ ray, neutron	leukemia	1×10^8^	*0.2	Shimizu et al. (1990)
II. Chronic exposure							
1) Internal radiation							
31	Mouse	CF1	PB ^90^Sr β-ray, injected	bone sarcoma	2×10^−5^	20	Finkel et al. (1959)
32	"	BC3F1	WB ^3^H β-ray, oral	thymic lymphoma	6.4×10^−7^	0.71	Yamamoto et al. (1998)
33	Rat	Long-Evans	PB ^131^I β-ray, injected	thyroid adenoma	1.7×10^−4^	3.3	Lee et al. (1982)
34	"	Sprague-Dawley	PB ^237^Np β-ray, inhaled	lung tumour	7×10^−4^	1	Dudoignon et al. (1999)
35	"	"	PB ^222^Rn α-ray, inhaled	"	3×10^−5^	0.19	Morlier et al. (1994)
36	"	Wister	PB ^238^PuO_2_ α-ray, inhaled	"	2.5×10^−4^	0.25	Sanders et al. (1977)
37	"	"	PB ^239^PuO_2_ α-ray, inhaled	"	3.4×10^−7^	0.05	"
38	"	"	PB ^244^CmO_2_ α-ray, inhaled	"	1.9×10^−5^	0.18	Sanders & Mahaffey (1978)
39	Dog	beagle	PB ^90^Sr β-ray, injected	bone sarcoma	6×10^−3^	30	Mays & Finkel (1980)
40	"	"	"	"	3.2×10^−3^	6.7	White et al. (1993)
41	"	"	PB ^144^Sr β-ray, inhaled	lung tumour	1.3×10^−5^	5	Hahn et al. (1999)
42	"	"	PB ^226^Ra α-ray, injected	bone sarcoma	5×10^−7^	0.9	White et al. (1994)
43	"	"	"	"	7×10^−7^	2	Rowland et al. (1973)
44	"	"	PB ^228^Ra β-ray, injected	"	2.8×10^−7^	5	"
45	Human	thorotrast patient	PB ^232^ThO_2_ α-ray, injected	liver cancer	1.1×10^−7^	*2	Anderson & Storm (1992)
46	"	dial painter	PB ^226^Ra + ^228^Ra α + β, oral	bone sarcoma	4.9×10^−7^	*10	Rowland et al. (1978)
2) External radiation							
47	Mouse	RFM/Un male	WB γ-ray	myeloid leukemia	3×10^−5^	1.5	Upton et al. (1970)
48	"	RFM/Un female	"	"	5×10^−6^	2.5	"
49	"	CBA/H	PB ^204^Tl β-ray, skin	skin tumour	2×10^−2^	16	Hulse et al. (1983)
50	"	ICR	PB ^90^Sr^−90^Y β-ray, skin	"	1.5 Gy/week, 6 months	*40	Ootsuyama & Tanooka (1991, 1993)
51	Dog	beagle	WB γ-ray, continuous	myeloproliferative disease	2×10^−6^	8.6	Thompson (1989)
52	Human	high radiation background area in India	"		1.3×10^−8^	*no cancer increase	Nair et al. (1999)
53	"	high radiation background area in China	"		5.7×10^−9^	*no cancer increase	Chen & Wei (1990)
Data added							
54	Dog	beagle	PB ^226^Ra α-ray	bone sarcoma	7×10^−7^	0.44	Raabe (1984)
55	Mouse	C.B-17	WB γ-ray	thymic lymphoma	5×10^−1^	1	Ishii-Ohba et al. (2007)
56	"	C57BL/6j	"	"	2×10^−5^	*>7	Ina et al. (2005)
Natural background radiation level					1.8×10^−9^		

a WB: Whole body radiation. PB: Partial body radiation.

* Not included in calculation for the regression line.

### Estimation of dose-rate

The values for the dose-rate were obtained from each published paper. For external radiation, the dose-rate was clearly presented in the literature either for whole body or partial body exposures. However, for internal radiation from radioactive nuclides, the estimation of dose-rate required assumptions and calculations depending on whether internal radioactive nuclides were distributed in the whole body or deposited partially in the target organ. Moreover, the radioactivity decayed with time and the radioactive nuclide was cleared from the body. In the present analysis, an average dose-rate was estimated from the total dose divided by the exposure time or, when a decay curve was available, an average dose-rate over the 70% decay time was taken. This calculation may have resulted in a lower estimate of dose-rate and a higher estimate of D_nt_, provided that the radiation dose given only in the first half of the exposure time was effective for tumour induction. However, correction for this gave little change in the plot of D_nt_ versus dose-rate on a bi-logarithmic scale.

## Results and discussion

**Figure 1:**
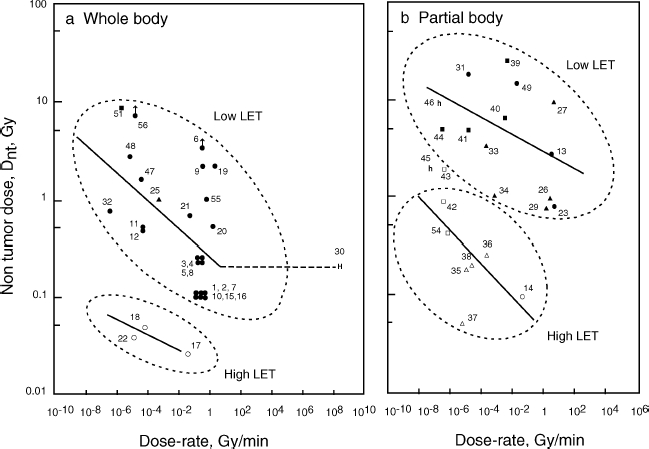
Non-tumour dose, D_nt_, plotted as a function of the dose-rate of radiation. (a) Whole body radiation. (b) Partial body radiation. Block symbols, low LET; open symbols, high LET. Mouse (•, ^); rat (▴, Δ); dog (▪, □); human, whole-body low LET (**H**); and human, partial body high LET (**h**). Arrows indicate D_nt_ higher. Numbers affixed to each point are data numbers (see Table I).

Numerical values for D_nt_ and corresponding dose-rates obtained from various tumour systems are listed in Table I. These values were divided into four groups, i.e., whole body irradiation with low LET and high LET radiation and partial body irradiation with low LET and high LET radiation, respectively. Figure 1 shows a plot of D_nt_ against dose-rate on a bi-logarithmic scale and regression lines fitted to the data for dose-rates below 1 Gy/min. A clear dose-rate dependence of D_nt_ is seen for the four exposure patterns.

For whole body irradiation with low LET radiation, D_nt_ increased when lowering the dose-rate below 1 Gy/min and became 20-fold higher at 10^−8^ Gy/min (Figure 1a). Only one point for humans was available for the high dose-rate 10^7^ Gy/min, based on the assumption that the A-bomb radiation was delivered in 1 msec. It appeared that D_nt_ is constant for dose-rates between 1 and 10^7^ Gy/min, as shown by the horizontal line in Figure 1a. For high LET irradiation of the whole body, there were few data available, but the dose-rate dependence of D_nt_ was seen at a level about 10- to 20-fold lower than for low LET radiation, although high LET radiation has been considered to have no dose-rate effect.

For partial body irradiation, the dose-rate dependence of D_nt_ was again seen for both low LET and high LET radiation (Figure 1b). Dose-response data for dose-rates higher than 10 Gy/min were not available in the literature. The D_nt_ level of partial body radiation was about 5- to 10-fold higher for low LET radiations and 3- to 5-fold higher for high LET radiations than those for whole body radiation.

At an extremely high dose-rate for whole body radiation, A-bomb survivor data (Shimizu et al. 1990) gave a D_nt_ of 0.2 Gy for leukemia mortality; while mouse data from nuclear detonation experiments at similar dose-rates showed a significant increase in pituitary and Harderian gland tumours at the same dose, 0.2 Gy (Furth et al. 1954). Consequently, humans seem to be more tolerant to radiation than mice and the regression lines drawn from animal data may under-estimate D_nt_ for humans. D_nt_ values, for partial body high-LET radiation to radium dial painters (Rowland et al. 1973, 1978) and thorotrast-injected patients (Anderson and Storm 1992), were much larger than those for experimental animals (Figure 1b), again indicating a higher radiation tolerance of humans. The other extreme case is the absence of thymic lymphoma induction in mice irradiated at 2 × 10^−5^ mGy/min with a total whole body dose of 7.2 Gy; whereas, acute radiation given in four fractions with the same total dose yielded a 90% tumour incidence (Ina et al. 2005), as was originally found in the early experiments of Kaplan and Brown (1952).

Fractionation of radiation dose at a fixed dose-rate within a defined time interval lowers cancer incidence, as shown in the induction of skin tumours by local irradiation in rats (Burns et al. 1973, 1975, 1993). However, fractionation necessarily involves repetitive irradiations, which results in a tumour-enhancing effect as seen for mouse thymic lymphoma induction (Kaplan and Brown 1952) and also in mouse skin tumour induction (Ootsuyama and Tanooka 1991). It should be noted that the repetitive treatment is efficient for chemical induction of tumours. This contradictory effect should be considered in analysing the dose-rate effect.

**Figure 2:**
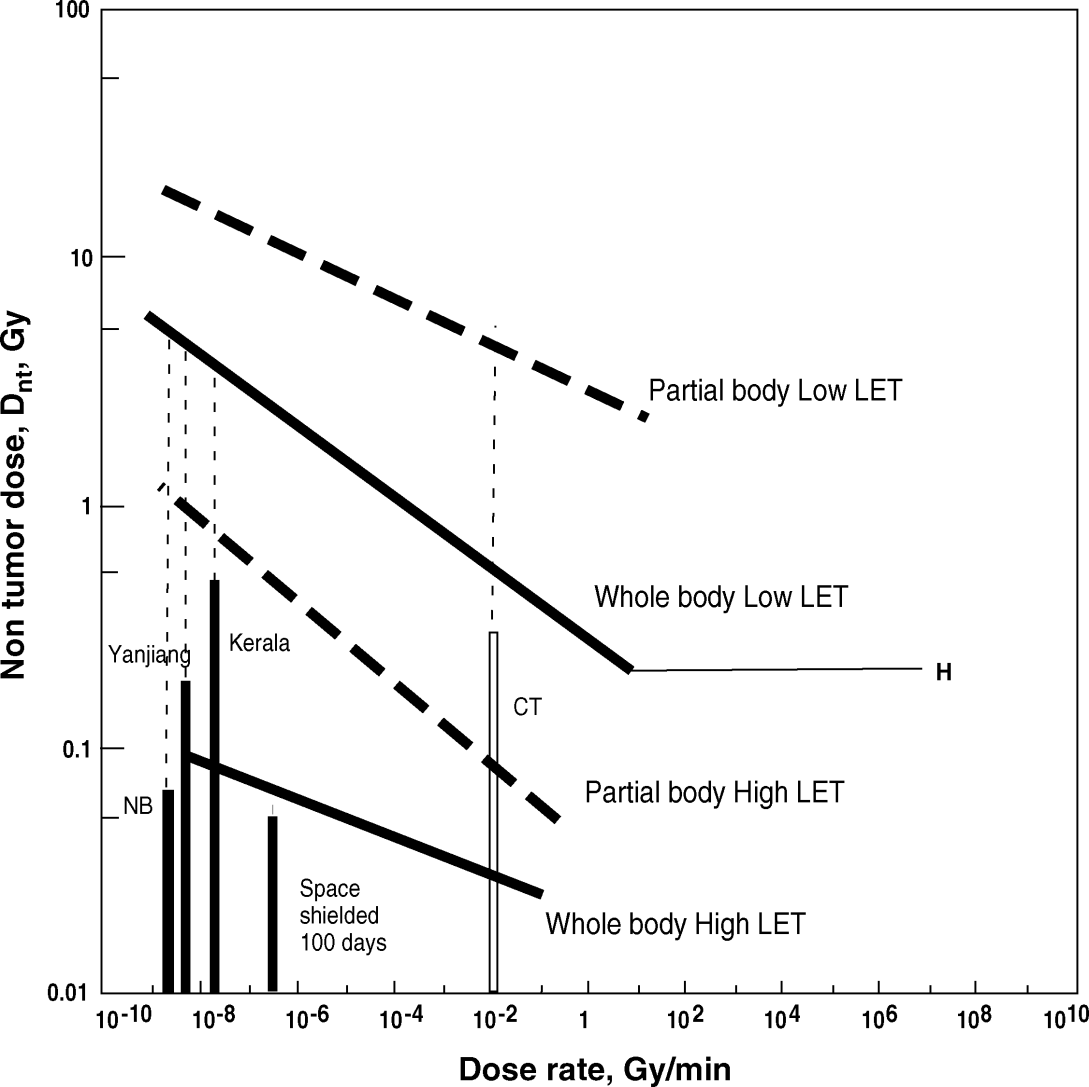
Summary of regression lines for non-tumour dose, D_nt_, versus dose-rate of radiation. Regression lines for dose-rate range from 10^−8^ to 1 Gy/min: whole body low LET, Y = 0.258 X^−0.141^, R^2^ = 0.320; whole body high LET, Y=0.0207 X^−0.0733^, R^2^ = 0.781; partial body low LET, Y = 2.69 X^−0.0857^, R^2^ = 0.147; partial body high LET; Y = 0.0439 X^−0.167^, R^2^ = 0.303. Bars: radiation doses received by residents in natural (NB) and high background areas in Kerala, India, and Yanjiang, China, over 70 years. CT: possible highest dose to patients under CT examination. Space: possible highest dose in space using a 10 g/cm^2^ shield for six months. Dotted vertical lines indicate the difference between exposure dose and corresponding D_nt_ value.

Figure 2 summarises the regression lines for the four exposure patterns. These four lines are thought to cover all possible radiation exposure cases and hopefully to serve as a measure of cancer risk for any exposure situation in the human environment. Total whole body radiation doses received over 70 years from the natural environment high background radiation areas in Kerala, India (Nair et al. 1999) and Yanjiang, China (Chen and Wei 1991) are much smaller than D_nt_ for the respective dose-rates in each district (Figure 2). The radiation dose to astronauts in space (Horneck et al. 2003) is also shown in Figure 2, indicating a value close to D_nt_ even with a radiation shield. The cancer risk of medical examination with computer tomography (CT) has been analysed on the basis of whole-body data of A-bomb survivors (Berrington de Gonzalez and Darby 2004); however, this risk should have been analysed on the basis of partial body data. The highest possible dose for CT was still far lower than the corresponding D_nt_. Recently, Tubiana et al. (in press) reported the dose response of second cancer incidence after radiation therapy with a D_nt_ of about 1 Gy based on a large number of patients. This study provides important data on human exposure to partial body low LET radiation.

There are differences in the radiation sensitivity of tumour induction, depending on the type of tumour and host sensitivity. D_nt_ is much smaller in repair-deficient mice compared to wild-type mice (Ishii-Ohba et al. 2007), indicating that the regression lines represent the wild-type character of the hosts. Currently, a large scale life-time exposure of mice to external γ rays with graded dose-rates from 1-800 mGy per 22 h a day (dose-rate: 7.5 × 10^−6^ − 6 × 10^−3^ Gy/min, total dose for 3 years: 1.1 - 876 Gy) together with control mice is being conducted and chromosome aberration data have been reported (Tanaka et al. 2009). Such experiments will give more accurate data for the effect of dose-rate on tumour induction. Further data will be needed to cover the whole dose-rate range for tumour induction.

## Summary

Meta-analysis of the non-tumour dose, D_nt_, of ionising radiation showed a clear dependence on dose-rate over a wide range for four exposure conditions, i.e., whole body irradiation with low LET or high LET radiation and partial body irradiation with low LET or high LET radiation. From the regression lines for the relation between dose-rate and D_nt_, a cancer risk or tolerance level of radiation could be estimated for a variety of exposure conditions. An apparent discrepancy in radiation-induced tumour data could be explained in terms of dose-rate.
